# The Analysis of Paratuberculosis Prevalence and Associated Performance Parameters in Dairy Cows from Xi’an City

**DOI:** 10.3390/vetsci12030243

**Published:** 2025-03-03

**Authors:** Xuejian Zhao, Qiang Liang, Haipeng Feng, Caixia Ru, Lei Wang, Kang Zhang, Jianxi Li

**Affiliations:** 1 Engineering & Technology Research Center of Traditional Chinese Veterinary Medicine of Gansu Province, Lanzhou Institute of Husbandry and Pharmaceutical Sciences, Chinese Academy of Agricultural Sciences, Lanzhou 730050, China; 15593323520@163.com (X.Z.); fenghp105@126.com (H.F.); wanglei@caas.cn (L.W.); 2 College of Veterinary Medicine, Gansu Agricultural University, Lanzhou 730070, China; 3 College of Pet Technology, Shandong Vocational Animal Science and Veterinary College, Weifang 261061, China; liangqiang3611@163.com; 4 Xi’an Caotan Farm Co., Ltd., Xi’an 710038, China; haipeng@mail.com

**Keywords:** paratuberculosis, dairy cow, serological testing, ELISA, performance parameters

## Abstract

The aim of this study was to understand the status of Mycobacterium paratuberculosis infection in a large-scale dairy farm in Xi’an city and evaluate the impact via a “quarantine + elimination” model of bovine paratuberculosis on the production performance, reproductive performance, and economic benefits in said dairy farm. The paratuberculosis antibodies from 4488 dairy cow sera were detected by an ELISA kit, complemented by a comprehensive analysis of milk production parameters, health metrics, reproductive indices, and pharmaceutical expenditures (2021–2024). The results indicated that the paratuberculosis prevalence in the dairy farm gradually reduced from 6.76% (2021) to 3.58% (2024). It was also found that the paratuberculosis prevalence among dairy cows increased progressively with the increase in parity until the fifth calving, after which a significant decline was observed. The reduction in infection rates in the herd was correlated with measurable improvements in milk quality metrics, including elevated milk fat and protein content, extended shelf stability, and decreased somatic cell counts in milk. In addition, the reproductive performance of the dairy cows relatively improved with the decrease in paratuberculosis prevalence; there was a relative improvement in the reproductive performance of the dairy cows, which mainly occurred by the time of pregnancy at the first service of the cows, while the number of monthly occurrences of endometritis, diarrhea, calving intervals, and inseminations decreased. Further data correlation analysis showed that daily milk volume was positively correlated with lactase persistence (95% CI: 0.247–0.753, *p* = 0.001) and peaked at the day of milk production (95% CI: 0.135–0.698, *p* = 0.008) but was negatively correlated with parity (95% CI: −0.783–−0.315). In addition, lactation time was positively correlated with 305-day milk volume (95% CI: 0.173–0.718, *p* < 0.004) and peaked at the day of milk production (95% CI: 0.265–0.761) but showed the opposite trend with the milk fat rate (95% CI: −0.633–−0.018, *p* = 0.040) and milk protein rate (95% CI: −0.738–−0.215, *p* = 0.002). Furthermore, milk loss was negatively correlated with peak milk production (95% CI: −0.758–−0.258, *p* = 0.001). Intriguingly, the cost of medications for diarrhea exhibited a downward trend over the past three years. Taken together, these findings confirmed the necessity to reduce the incidence of *Mycobacterium avium* subsp. paratuberculosis in dairy cows and serve as a guide for the future successful and gradual eradication of paratuberculosis in Chinese dairy cow farms.

## Simple Summary

The diarrhea incidence of dairy cows in a large-scale dairy farm in Xi’an city was high, resulting in a significant increase in drug costs for prevention and therapeutics with limited clinical treatment effects. It was confirmed to be a paratuberculosis infection after being diagnosed by an ELISA test and combined with clinical manifestations. Subsequently, a measure of the “quarantine + elimination” model of bovine paratuberculosis was taken to control the disease on a yearly basis. The specific milk production parameters, reproductive indices, and related prevention and control expenditure records for dairy cows from 2021 to 2024 were analyzed by using dairy cow management software. The results showed that the “quarantine + elimination” model of bovine paratuberculosis effectively reduced the seroprevalence of paratuberculosis. Meanwhile, with the elimination and quarantine of paratuberculosis-positive cattle over the past three years, milk production parameters and reproductive index scores in cows relatively improved, and the ineffective expenditure in the farm was greatly reduced. This research emphasizes the necessity of the “quarantine + elimination” model of bovine paratuberculosis to control the prevalence of paratuberculosis in dairy cows and improve ranch benefits.

## 1. Introduction

Bovine paratuberculosis (PTB), also known as John’s disease, is a chronic gastrointestinal infectious disease of ruminants caused by *Mycobacterium avium* subsp. paratuberculosis (MAP) [1,2]. PTB is classified as a Class B animal disease by the World Organization for Animal Health and as a Class II animal disease in China. Diagnostic approaches to PTB detection are different for dairy cows; large-scale bovine PTB is usually diagnosed by ELISA using blood or milk samples, and fecal samples are often used in small-scale PTB studies (fecal suspension followed by supernatant). MAP is a small Gram-positive bacillus that is resistant to cold, dryness, certain acidic environments, and ultraviolet radiation, which is widely prevalent around the world [3]. MAP strains are divided into three subtypes, namely type I (sheep type), type II (cattle type), and type III (pigmented type), based on culture characteristics and pathogenicity; the bovine strain, in particular, infects not only cows but other species as well [4,5,6]. In addition, it is an intracellular, parasitic acid-fast-staining-positive bacterium. The use of antibiotics to treat this only relieves clinical symptoms and does not completely eradicate the disease. Previous research indicated that cow farms suffered serious economic losses due to the fact that the incubation period of bovine PTB ranges from several months to several years, with the total annual economic loss per cow ranging between USD 21 and 79 in the US [7]. Currently, no available treatment drugs and vaccines have been reported for this disease either.

Previous studies report that PTB infection is highly associated with health and fertility issues in dairy cows, particularly in their productivity, reproductive performance, lameness incidence, and lying behavior during peak lactation [8,9]. MAP infections have seriously damaged milk production, calving intervals, and milk fat/milk protein content in infected cows [10,11]. PTB is transmitted through the placental barrier to newborn calves either horizontally or vertically, thus increasing the risk of transmission and spreads in the herd [12]. Therefore, the best way to eradicate the disease is to detect, isolate, and eliminate infectious dairy cows.

In recent years, PTB infection has become widely prevalent around the world, not only affecting domestic and wild ruminants globally but also affecting non-ruminants and carnivores, among others [13,14,15]. Previous studies indicate that the true herd-level prevalence in dairy cattle herds is estimated to be 19.6%, 9.8%, 6.3%, 3.6%, 9.4%, and 18.4% in Hungary [16], Brazil [17], Chile [18], Switzerland [19], Italy [20], and Spain [21], respectively. For dairy animals in the USA, the prevalence of PTB infection has been reported to reach up to 5–10%, and it is 33% in herds [22]. In addition, a report indicated that the PTB prevalence in infected cows increased with age and somatic cell count according to MAP test results, while milk yield, milk protein, and milking days are negatively correlated with MAP infection [23]. Meanwhile, the incidence of PTB also exhibited an up-regulated trend in China [24,25]. *Mycobacterium paratuberculosis avium* subspecies is structurally similar to *Mycobacterium tuberculosis antigens* and is prone to exhibit immune cross-reaction, so the discrimination method must be accurately grasped. Therefore, the study aims to investigate the correlation of MAP infection with the milk production performance, health status, and reproductive performance of dairy cows in Xi’an city (from 2021 to 2024), with the objective of providing data support for the clinical eradication and prevention strategies for bovine PTB.

## 2. Materials and Methods

### 2.1. Serum Sample

This work was conducted on a dairy farm (stock: 3250 cows) from July 2021 to June 2024. All cows from the dairy farm were vaccinated according to the normal immunization schedule. Cows were fed a total mixed ration based on corn silage, milked three times a day in a fixed milking parlor, and had free access to water. Blood with a volume of 3–5 mL was collected from the jugular vein of two-year cows and then centrifuged at 2500 rpm for 10 min (min). Next, supernatants were collected and stored at −20 °C until testing.

### 2.2. Reagents

The commercial bovine Mycobacterium paratuberculosis indirect ELISA antibody detection kit was purchased from Shenzhen Yirui Biotechnology Co., Ltd. (Shenzhen, China) (Cat number: YR2AD04A01).

### 2.3. Methods

#### 2.3.1. The ELISA Detection for Bovine PTB Antibody

The serum samples and reagents were equilibrated to room temperature before use. All testing procedures were performed rigorously in accordance with the manufacture’s protocol. For test validity, the following criteria were required: the mean OD value of positive control must be ≥0.8; the ratio of positive control OD to negative control OD (P/N ratio) must be ≥5:1 (calculated as mean positive control OD divided by mean negative control OD). Then, sample interpretation was determined by calculating the sample-to-positive ratio (S/P) using the formula S/P = (Sample OD − Mean Negative Control OD)/(Mean Positive Control OD − Mean Negative Control OD). (S/P) ≥ 0.3 indicated antibody positivity, and S/P < 0.3 indicated antibody negativity.

#### 2.3.2. The Determination of Milk Production Performance Indicators in Cows

A statistical comparison was conducted to evaluate the change in daily milk production of lactating dairy cows between 2021 and 2024. Milk samples were collected monthly and analyzed, including milk fat percentage, milk protein percentage, the ratio of fat to protein, somatic cell count, and somatic cell fraction in milk. The urea nitrogen content, lactation time, duration, 305-day milk yield, milk loss, peak milk production, and peak day were also analyzed. All parameters were subsequently sent to the Dairy Cow Production Performance Measurement Center for standardized measurement.

#### 2.3.3. The Determination of Health Status and Reproduction-Related Indicators from Cows

During the lactation period of dairy cows, the monthly incidence of endometritis and mastitis, calving interval, artificial insemination number, the pregnancy at the first service, and parity of the entire herd were counted yearly.

#### 2.3.4. Bovine Tuberculosis Testing

When PTB occurred, combination monitoring for bovine tuberculosis infection was immediately carried out by using two methods at the same time. According to the “Animal Quarantine Operation Procedures” from the Ministry of Agriculture in China, domestic purified tuberculin was used for intradermal testing. The local heat, pain, and swelling were observed after 72 h of infection, and then the thickness was further measured. If the result showed an obvious inflammatory reaction, a skin thickness of 4 mm was positive. Suspected reaction cattle were retested 30 days after the first test. If the result was still suspicious after 30–45 days, it was still suspected positive. The ELISA detection method for bovine tuberculosis antibody was similar to that for bovine PTB antibody.

#### 2.3.5. Statistics on Veterinary Drug Expenditure

Statistics were collected on the expenses of various vaccines administered on the farm each year, expenses on endometritis, mastitis, postpartum recovery care drugs, various medical consumables, and diarrhea treatment drugs, comprising Pulsatilla powder, baytril, penicillin, and gentamicin.

#### 2.3.6. Measures for Culling PTB-Positive Cattle

The ELISA-positive cattle were isolated in separate cattle pens immediately and retested with the ELISA kit to eliminate false positive interference. If the retest result was still positive, professionals used electric shock to immediately paralyze the central nervous system of the cattle under professional guidance from local government, causing them to lose consciousness quickly and stop breathing to minimize the pain of cattle. Next, the carcass was incinerated to ensure that it was completely carbonized after being slaughtered, and then it was buried deep in a designated location and recorded. An initial inspection and secondary inspection were carried out once a year, and all positive cattle in the retest were slaughtered.

#### 2.3.7. Statistical Analysis

Microsoft Excel 2016 software was used for the preliminary processing of the experimental data, and the results were expressed as “mean ± standard error”. SPSS statistics 27.0 software was used to analyze the significance of the differences. One-way analysis of variance was performed on the measurement data, and χ^2^ was performed on the percentage data. For the test, *p* < 0.05 was set as the standard for evaluating differences.

## 3. Results

### 3.1. The Antibody Level of PTB in Dairy Cows over the Past Three Years

As shown in Table 1, there is a significant difference between the prevalence of PTB in 2023–2024 (3.58%) and 2021–2022 (6.76%, χ^2^ = 14.553, *p* < 0.001) in dairy cows. The prevalence of PTB in the 2023–2024 year (3.58%) and 2022–2023 year (6.83%) showed an extremely significant difference (χ^2^ = 15.075, *p* < 0.001) in dairy cows. There was no significant difference in PTB prevalence between the 2021–2022 year (6.67%) and 2022–2023 year (6.83%, χ^2^ = 0.007, *p* > 0.05).

### 3.2. The Correlation Analysis of PTB Prevalence from Various Parities in Dairy Cows

The ELISA results exhibited that the prevalence of PTB in dairy cows increased with the increase in parity, until it dropped significantly at the fifth parity. The prevalence showed extremely significant differences between the zeroth parity and the second parity (χ^2^ = 9.952, *p* = 0.002). And the third parity (χ^2^ = 14.292, *p* < 0.001), the fourth pregnancy (χ^2^ = 21.472, *p* < 0.001), and the fifth pregnancy and above (χ^2^ = 6.934, *p* = 0.008) showed extremely significant differences. Furthermore, the prevalence showed extremely significant differences between the first pregnancy and the second pregnancy (χ^2^ = 10.596, *p* = 0.001), the third pregnancy (χ^2^ = 18.807, *p* < 0.001), and the fourth pregnancy (χ^2^ = 29.584, *p* < 0.001). A consistent trend was exhibited in the second parity and third parity (χ^2^ = 7.653, *p* = 0.006) and the fourth and fifth or above (χ^2^ = 4.143, *p* = 0.042), as shown in Table 2.

### 3.3. Changes in Indicators Related to Milk Production Performance of Dairy Cows with Different PTB Prevalences over the Past Three Years

As shown in Table 3, milk fat percentage and maximum milk yield were significantly higher in the 2022–2024 year than the 2021–2022 year (*p* < 0.05), milk protein percentage was significantly higher in the 2022–2023 year than the 2021–2022 year (*p* < 0.05), somatic cell counts and milk losses decreased significantly year by year (*p* < 0.05), somatic cell counts in milk in the 2023–2024 year were significantly lower than in the 2022–2024 year (*p* < 0.05), 305-day milk yield was significantly higher in the 2022–2024 year than the 2021–2022 year (*p* < 0.05), somatic cell fraction in milk was significantly lower in the 2023–2024 year than in the 2021–2023 year (*p* < 0.05), and peak day was significantly lower in 2024 than 2022 (*p* < 0.05). These results indicated that the PTB prevalence reduction contributes to improving milk production performance and milk nutrition in dairy cow herds.

### 3.4. Changes in Reproductive Performance Index in Dairy Cows with Different Incidence Rates of PTB over the Past Three Years

As shown in Table 4, the monthly incidence of mastitis was significantly higher (*p* < 0.05) in the 2023–2024 year than in the 2021–2022 year. The number of inseminations was significantly lower (*p* < 0.05) in the 2023–2024 year than in the 2022–2024 year, and the calf production number was significantly higher (*p* < 0.05) in the 2023–2024 year than in the 2022–2023 year. In addition, the monthly incidence of endometritis and calving intervals had no difference (*p* > 0.05) over the past three years, and both showed a decreasing trend year by year, which indicated that PTB prevalence reduction significantly improved the reproductive performance in dairy cows.

### 3.5. The Prevalence of Tuberculosis in Dairy Cows from 2021 to 2024

Based on our detection results, the prevalence of bovine tuberculosis quarantine from 2021 to 2024 was lower than 0.5%. PTB testing was conducted on tuberculosis-positive cattle, which displayed that the repetition rate of tuberculosis and PTB positivity in the 2021–2022 year was 85.71%, as well as completely overlapping with the 2022–2023 year and 2023–2024 year, as shown in Table 5. These results confirmed that *Mycobacterium avium* subspecies PTB increased the risk of tuberculosis infection in cattle. Finally, we performed ELISA detection on bovine tuberculin-positive cattle, and all results were negative, as shown in Table 6.

### 3.6. The Prevention and Control Expenditures in the Dairy Farm over the Past Three Years

By investigating the annual expenditures for disease prevention and control in the dairy farm, we found that the monthly expenses in the 2022–2023 and 2023–2024 years were significantly lower than the 2021–2022 year (*p* < 0.05), including veterinary drugs, vaccines, and medical consumables, as shown in Table 7. It is worth noting that the monthly expenses for diarrhea drugs showed a decreasing trend over the past three years (*p* < 0.05), which indicated that the prevalence of PTB reduction significantly alleviated diarrhea occurrence in dairy herds and significantly reduced non-essential expenses on farms.

## 4. Discussion

PTB has become widespread in dairy farms, causing serious damage to dairy cows’ milk production and making the adoption of prevention and control measures difficult. According to relevant reports in China, the PTB antibody prevalence in dairy cows was 9.25% in Beijing, 5.73% in Gansu, 17.44% in northern Xinjiang, 14.10–86.67% in Shandong and Ningxia, and 4.31% in Heilongjiang Province. The same results were discovered in Pakistan and Ecuador, which exhibited that the prevalence of PTB was 3.8% and 25%. The above results confirmed that PTB is prevalent in major dairy farming areas in China, and there are differences in various regions of each country around the world. An analysis of the survey data showed that the prevalence of bovine PTB in a farm from Xi’an city has dropped from 6.76% to 3.58% as a result of continuous monitoring and elimination over the past three years. In addition, a previous study reported that herd scales, the purchase of cattle from unknown MAP infection status, and different feeding methods for calves play an important role in the MAP positivity rate of an entire farm [26]. Therefore, we can pay sufficient attention to preventing and controlling MAP infection, especially in areas where the prevalence of MAP among cattle herds is high.

Our results found that the PTB prevalence in dairy cows was gradually increased with the increase in parity, which was inconsistent with the results of Bates A in New Zealand, because it was higher in primiparous dairy cows than in multiparous dairy cows in two out of three years according to PTB prevalence statistics [27]; the difference might be associated with the immune system and the increasing parturition number. The results also exhibited that the milk fat content increased from 3.61% to 4.11%, exceeding the national standard milk level at 4%. The milk protein content increased from 3.28% to 3.41%, the ratio of fat to protein in milk increased by 0.13%, the somatic cell count in milk decreased by 10.2 × 10^3^ mL^−1^, the somatic cell count in milk decreased by 0.52 × 10^3^ mL^−1^, the milk loss decreased by 0.38 kg, the peaked milk production increased by 0.11 kg, and the duration was extended by 1.87 days. The number of monthly diarrhea occurrences decreased from 18.75 to 10.33 cows, the number of artificial inseminations decreased by 0.32 cows, the number of monthly endometritis occurrences decreased by 0.16 cows, the calving interval decreased by 4.43 days, the pregnancy at the first service increased by 0.35%, and the number of monthly mastitis occurrences increased by 16.9 cows. The above results indicated that continuous monitoring and active elimination of PTB-antibody-positive cows not only had a positive effect on milk quality, but also significantly reduced the occurrence of some reproductive diseases, including endometritis and mastitis, which is consistent with the previous results [28,29,30]. The change in daily milk yield was not obvious in the 2022–2023 year. Meanwhile, we speculated that the decrease in the 305-day milk yield, peak day, and lactation time of the herd may be correlated to the milking disinfectant products or the non-standard disinfection operations of milking workers [31]. In addition, the monthly occurrence of mastitis and the trend of the milk protein rate are consistent, which may be associated with the increasing number of milking times for cows during the peaking lactation period to improve economic benefits in large-scale dairy farms, thereby increasing the mastitis infection rate by promoting the permeability of mammary epithelial cells and the blood–milk barrier, resulting in an increase in the milk protein rate. Meanwhile, the milk fat rate and milk protein rate of high-yielding dairy cows in actual measurements were reduced due to the influence of the “dilution effect” [32]. Laszlo et al. reported that the somatic cell count in positive cow’s milk (35.8%) was higher than that of negative cows on average [33], which is basically consistent with the results in our study showing that the somatic cell count in milk in the 2022–2024 year was higher (45.0%) than that in the 2023–2024 year. The annual increase in urea nitrogen in milk may be caused by the fact that breeders increased the protein content in the feed to improve milk quality, and in a previous study, it was confirmed that controlling the quality of milk consumed by calves cannot prevent the spread of MAP infection [34]. Therefore, changing feed formulations with parity-specific protein levels could be considered to optimize protein utilization efficiency in future breeding programs.

Antibodies directed against MAP epitopes could be detected in milk and other specimens as well as in serum. Cattle that are infected with tuberculosis will continue to excrete bacteria through feces. Currently, the specific diagnostic method for quarantine monitoring of cattle herds is the tuberculin test, and there are no other diagnostic methods for relatively easy performance at a low cost. Mycobacterium PTB can also produce tuberculin, and the PTB test was conducted on tuberculosis-positive cattle from 2021 to 2024. The result showed that the repetition rate of tuberculosis and PTB-positive cattle numbers in the 2021–2022 year was 85.71%, and it completely overlapped with that in the 2022–2024 year, indicating the accuracy of the bovine PTB test. In addition, the monthly expenditure on veterinary drugs, vaccines, and medical consumables in the 2023–2024 year was less than that in the 2021–2022 year, and the monthly expenditure on diarrhea-related drugs decreased year by year. In an analysis of economic losses due to reduced milk production and reproductive performance associated with bovine PTB in Switzerland, the total annual loss of PTB-positive cows was USD 1.3 × 10^9^ [35]. It is worth pointing out that this finding was not attributed to the efficacy of diarrhea medications, but rather to preventing and controlling the disease through a combination of regular quarantine, isolation, and culling, as well as effective biosecurity management [36]. It is worth noting that farms will need to continue to invest in regular tests and culling when eradicating bovine PTB through testing and culling alone. Therefore, the cost-effectiveness of a herd-scale testing and selective culling strategy is measured by the number of positive cows on a farm, maximizing the cost-saving investment. In public health contexts, bovine PTB (*Mycobacterium avium* subspecies PTB) presents zoonotic transmission risks through the food chain, posing significant public health risks, including its potential association with human rheumatoid arthritis [37,38,39]. Consequently, implementing comprehensive control strategies for bovine PTB not only serves as a prerequisite for ensuring sustainable dairy production systems but also represents a critical intervention for safeguarding public health security.

In summary, bovine PTB is a disease that seriously endangers the healthy breeding of dairy cows, and it is highly contagious and lacks available treatment drugs, thereby resulting in a significant reduction in dairy cow production. Meanwhile, the almost ineffective treatment costs will cause great economic losses for farms. Therefore, there is an urgent need to strictly control the spread of the disease by conducting quarantine, isolation, and elimination measures.

## 5. Conclusions

Our research investigated the PTB prevalence in dairy cows from a large-scale farm in Xi’an city from July 2021 to June 2024 and clarified that measures such as detection, isolation, and elimination can effectively reduce the prevalence of PTB in dairy cows, thereby improving the quality of milk production and restoring the normalization of breeding-related indicators. Meanwhile, monitoring the PTB antibody significantly reduced expenses on various veterinary drugs and medical consumables, especially diarrhea drugs for dairy cows, further confirming the necessity to support the control of MAP in dairy cows and avoid the significant production losses associated with it.

## Figures and Tables

**Table 1:** The result of the PTB test.

Year	2021–2022	2022–2023	2023–2024
The number of tested cows/cows	1583	1566	1339
The negative number/cows	1476	1459	1291
The positive number/cows	107	107	48
The prevalence/%	6.76	6.83	3.58

**Table 2:** The correlation analysis of PTB prevalence in dairy cows from various parities.

Parity	0	1	2	3	4	≥5
The number of tested cows/cows	310	1628	1236	722	335	257
The negative number/cows	304	1565	1155	663	298	241
The positive number/cows	6	63	81	59	37	16
The prevalence/%	1.94	4.03	6.55	8.17	11.04	6.23

**Table 3:** The change in milk production performance-related indicators over the past three years.

Indicators	2021–2022	2022–2023	2023–2024
Daily milk intake/L	36.5 ± 1.41	36.20 ± 0.93	36.13 ± 1.35
Milk fat percentage/%	3.61 ± 0.58 ^b^	4.30 ± 0.32 ^a^	4.11 ± 0.24 ^a^
Milk protein rate/%	3.28 ± 0.12 ^b^	3.45 ± 0.07 ^a^	3.41 ± 0.12
Fat-to-protein ratio in milk/%	1.09 ± 0.17 ^a^	1.24 ± 0.99 ^b^	1.21 ± 0.07 ^b^
The somatic cell count in milk/(10^3^·mL^−1^)	27.10 ± 6.80 ^a^	21.91 ± 2.30 ^b^	16.90 ± 2.64 ^c^
The fraction of somatic cells in milk	2.95 ± 0.41 ^a^	2.72 ± 0.23 ^a^	2.43 ± 0.64 ^b^
Urea nitrogen (mg/dL)	15.12 ± 1.49	14.99 ± 3.19	16.73 ± 2.20
Lactation time/d	173.33 ± 16.56	168.71 ± 13.66	163.16 ± 12.61
Sustainability/d	98.63 ± 2.18	99.51 ± 4.42	100.50 ± 2.26
305-day milk yield (kg)	10991.56 ± 94.27 ^a^	10784.67 ± 214.91 ^b^	10555.33 ± 122.87 ^b^
The loss of milk (kg)	0.77 ± 0.21 ^a^	0.60 ± 0.09 ^b^	0.48 ± 0.07 ^c^
The peak milk production (kg)	44.78 ± 0.63 ^b^	45.60 ± 0.99 ^a^	45.44 ± 0.37 ^a^
The peak day of milk production (d)	72.11 ± 7.70 ^a^	65.56 ± 2.60	63.33 ± 7.71 ^b^

Note: Data with different letters in the same column indicate significant differences (*p* < 0.05), while data with the same letters or no letters in the same column indicate no significant differences (*p* > 0.05).

**Table 4:** The determination of health and reproduction-related indicators from dairy cows over the past three years.

Indicator	2021–2022	2022–2023	2023–2024
Monthly incidence of endometritis/time	0.41 ± 0.79	0.66 ± 1.15	0.25 ± 0.62
Monthly incidence of mastitis/time	83.16 ± 16.35 ^b^	113.83 ± 30.54 ^a^	99.25 ± 25.80
Monthly diarrhea occurrence/time	18.75 ± 6.23 ^a^	15.66 ± 6.11 ^a^	10.33 ± 4.92 ^b^
Calving interval/days	401.67 ± 12.06	397.00 ± 5.00	397.33 ± 6.66
Number of artificial inseminations/time	1.95 ± 0.17 ^a^	1.89 ± 0.25 ^a^	1.63 ± 0.17 ^b^
The pregnancy rate at the first service/%	49.68 ± 6.52	50.98 ± 8.58	49.93 ± 11.49
Parity/birth	2.13 ± 0.07	2.10 ± 0.05 ^b^	2.16 ± 0.07 ^a^

Note: Data in the same industry with different letters on the shoulder indicate significant differences (*p* < 0.05), while data with the same letters or no shoulder indicate no significant differences (*p* > 0.05).

**Table 5:** The tuberculin detection results and TB number over the past three years.

Year	2021–2022	2022–2023	2023–2024
The number of tested cows/cows	3097	2804	2619
The positive number/cows	14	7	4
The prevalence rate/%	0.45	0.25	0.15
The positive number of tuberculosis and PTB/cows	12	7	4
The co-prevalence of tuberculosis and PTB/%	85.71	100	100

**Table 6:** The ELISA detection results and TB number over the past three years.

Year	2021–2022	2022–2023	2023–2024
Tuberculin test positive cattle/cows	14	7	4
ELISA-positive cattle/cows	0	0	0

**Table 7:** The expenditures for production and disease prevention over the past three years.

Item	2021–2022	2022–2023	2023–2024
Monthly expenses for veterinary drugs, vaccines, and medical consumables/CNY 10,000	40.65 ± 5.41 ^a^	28.61 ± 10.22 ^b^	27.00 ± 2.88 ^b^
Monthly cost for diarrhea medication/CNY 10,000	2.16 ± 1.61 ^a^	1.05 ± 0.27 ^b^	0.38 ± 0.21 ^c^

Note: Data in the same row with different letters indicate significant differences (*p* < 0.05), and data with the same letters indicate no significant differences (*p* > 0.05).

## Data Availability

The data that support the findings of this study are available on request from the corresponding author.

## References

[1] Nunney E., Crotta M., Bond K., van Winden S., Green M., Guitian J. (2023). Dataset on risk factors for seroconversion against *Mycobacterium avium* subspecies paratuberculosis in dairy cows. Data Brief.

[2] Lu N., Niu Y.-L., Song Y., Zhang D.-D., Jiang J., Wei J., Geng H.-L., Cao H. (2023). Preventive veterinary medicine106043-106043.Na L, YaLing N, Yang S; et al. Prevalence of paratuberculosis in cattle in China: A systematic review and meta-analysis. Prev. Vet. Med..

[3] Fechner K., Dreymann N., Schimkowiak S., Czerny C.-P., Teitzel J. (2019). Efficacy of dairy on-farm high-temperature, short-time pasteurization of milk on the viability of *Mycobacterium avium* ssp. paratuberculosis. J. Dairy Sci..

[4] Jessu A., Cochard T., Burtin M., Crapart S., Delafont V., Samba-Louaka A., Biet F., Moyen J.-L., Héchard Y. (2024). Extensive environmental survey of free-living amoebae and their elusive association with *Mycobacterium bovis* or *Mycobacterium avium* subsp. paratuberculosis. FEMS Microbiol. Ecol..

[5] Sohal J.S., Singh S.V., Singh P.K. (2009). On the evolution of ‘Indian Bison type’ strains of *Mycobacterium avium* subspecies paratuberculosis. Microbiol. Res..

[6] Hosseiniporgham S., Biet F., Ganneau C., Bannantine J.P., Bay S., Sechi L.A. (2021). A Comparative Study on the Efficiency of Two *Mycobacterium avium* subsp. paratuberculosis (MAP)-Derived Lipopeptides of L3P and L5P as Capture Antigens in an In-House Milk ELISA Test. Vaccines.

[7] Whittington R., Donat K., Weber M.F., Kelton D., Nielsen S.S., Eisenberg S., Arrigoni N., Juste R., Sáez J.L., Dhand N. (2019). Control of paratuberculosis: Who, why and how. A review of 48 countries. BMC Vet. Res..

[8] Rossi G., Grohn Y., Schukken Y., Smith R. (2017). The effect of *Mycobacterium avium* ssp. paratuberculosis infection on clinical mastitis occurrence in dairy cows. J. Dairy Sci..

[9] Martins E., Oliveira P., Oliveira B., Mendonça D., Niza-Ribeiro J. (2018). Association of paratuberculosis sero-status with milk production and somatic cell counts across 5 lactations, using multilevel mixed models, in dairy cows. J. Dairy Sci..

[10] Charlton G.L., Bleach E.C., Rutter S.M. (2019). Cows with paratuberculosis (Johne’s disease) alter their lying behavior around peak lactation. J. Dairy Sci..

[11] Wiszniewska-Łaszczych A., Liedtke K.G., Szteyn J.M., Lachowicz T. (2020). The Effect of *Mycobacterium avium* subsp. Paratuberculosis Infection on the Productivity of Cows in Two Dairy Herds with a Low Seroprevalence of Paratuberculosis. Animals.

[12] Mary G. (2020). *Mycobacterium avium* Paratuberculosis: A Disease Burden on the Dairy Industry. Animals.

[13] Cunha M.V., Rosalino L.M., Leão C., Bandeira V., Fonseca C., Botelho A., Reis A.C. (2020). Ecological drivers of *Mycobacterium avium* subsp. paratuberculosis detection in mongoose (*Herpestes ichneumon*) using IS900 as proxy. Sci. Rep..

[14] Beinhauerova M., Slana I. (2023). Utilisation of Actiphage in combination with IS900 qPCR as a diagnostic tool for rapid determination of paratuberculosis infection status in small ruminant herds. J. Vet. Res..

[15] Šmiga Ľ., Čurlík J., Lazár P., Drážovská M., Iglódyová A., Barbušinová E., Novotný J., Konjević D., Bhide M., Mojžišová J. (2020). Detection of *Mycobacterium avium* subsp. paratuberculosis in Slovakian wildlife. Pol. J. Vet. Sci..

[16] Vass-Bognár B., Khol J.L., Baumgartner W., Fornyos K., Papp M., Abonyi-Tóth Z., Bakony M., Jurkovich V. (2024). Investigating the Prevalence of Paratuberculosis in Hungarian Large-Scale Dairy Herds and the Success of Control Measures over Four Years. Animals.

[17] Camilo S.L.O., Fritzen J.T.T., Pereira U.d.P., Mota R.A., Alfieri A.A., Lisbôa J.A.N. (2022). Presence of antibodies against *Mycobacterium avium* subspecies paratuberculosis in Brazilian high-producing dairy herds. Braz. J. Microbiol..

[18] Verdugo C., Valdes M.F., Salgado M. (2020). Herd level risk factors for *Mycobacterium avium* subsp. paratuberculosis infection and clinical incidence in dairy herds in Chile. Prev. Vet. Med..

[19] Ottardi M., Lechner I., Wang J., Schmitt S., Schneeberger M., Schmid R.M., Stephan R., Meylan M. (2024). Seroprevalence of *Mycobacterium avium* subsp. paratuberculosis in Swiss dairy herds and risk factors for a positive herd status and within-herd prevalence. Front. Vet. Sci..

[20] Noemi M., Elisa R., Cecilia T., Francesca B., Fabrizio P. (2024). Long-term outcomes of the Italian *Mycobacterium avium* subspecies paratuberculosis control programme for dairy cattle. Vet. Rec..

[21] Stefanova E.P., Paz-Sánchez Y., Quesada-Canales Ó., Quintana-Montesdeoca M.d.P., Monteros A.E.d.L., Ramírez A.S., Fernández A., Andrada M. (2024). Caprine Paratuberculosis Seroprevalence and Immune Response to Anti-*Mycobacterium avium* Subspecies paratuberculosis Vaccination on the Canary Islands, Spain. Vet. Sci..

[22] Machado G., Kanankege K., Schumann V., Wells S., Perez A., Alvarez J. (2018). Identifying individual animal factors associated with *Mycobacterium avium* subsp. paratuberculosis (MAP) milk ELISA positivity in dairy cattle in the Midwest region of the United States. BMC Vet. Res..

[23] Rehman A.U., Javed M.T., Ahmed I., Saeed M.A., Ehtisham-Ul-Haque S., Rafique M.K., Sikandar A., Nasir A., Ahmad L., Kashif M. (2024). Serological and epidemiological investigation of *Mycobacterium avium* subspecies paratuberculosis in bovines in Pakistan. Anim. Biosci..

[24] Li L., Katani R., Schilling M., Kapur V. (2016). Molecular Epidemiology of *Mycobacterium avium* subsp. paratuberculosis on Dairy Farms. Annu. Rev. Anim. Biosci..

[25] Cheng Z., Liu M., Wang P., Liu P., Chen M., Zhang J., Liu S., Wang F. (2020). Characteristics and Epidemiological Investigation of Paratuberculosis in Dairy Cattle in Tai’an, China. BioMed Res. Int..

[26] Gaffuri A., Barsi F., Magni E., Bergagna S., Dellamaria D., Ricchi M., De Paolis L., Galletti G., Arrigoni N., Lorenzi V. (2023). A Survey in 33 Northern Italy Dairy Goat Farms. Animals.

[27] Andrew B., Rory O., Simon L., Frank G. (2019). Control of *Mycobacterium avium* subsp. paratuberculosis infection on a New Zealand pastoral dairy farm. BMC Vet. Res..

[28] Pritchard T.C., Coffey M.P., Bond K.S., Hutchings M.R., Wall E. (2017). Phenotypic effects of subclinical paratuberculosis (Johne’s disease) in dairy cattle. J. Dairy Sci..

[29] Chen Y., Hou L., Khalid A.K., Robertson I.D., Zhao Y., Chen X., Guo A. (2024). Individual- and Herd-Level Milk ELISA Test Status and Incidence for Paratuberculosis in Hubei Province, China. Vet. Sci..

[30] Griss S., Knific T., Buzzell A., Carmo L.P., Schüpbach-Regula G., Meylan M., Ocepek M., Thomann B. (2024). A scoping review on associations between paratuberculosis and productivity in cattle. Front. Vet. Sci..

[31] Fitzpatrick S.R., Garvey M., Flynn J., O’brien B., Gleeson D. (2021). The effect of disinfectant ingredients on teat skin bacteria associated with mastitis in Irish dairy herds. Ir. Vet. J..

[32] Ozsvari L., Harnos A., Lang Z., Monostori A., Strain S., Fodor I. (2020). The Impact of Paratuberculosis on Milk Production, Fertility, and Culling in Large Commercial Hungarian Dairy Herds. Front. Vet. Sci..

[33] Steuer P., Tejeda C., Moroni M., Verdugo C., Collins M.T., Salgado M. (2021). Attempted Control of Paratuberculosis in Dairy Calves by Only Changing the Quality of Milk Fed to Calves. Animals.

[34] Rieger A., Meylan M., Hauser C., KnubbenSchweizer G. (2021). Meta-analysis to estimate the economic losses caused by reduced milk yield and reproductive performance associated with bovine paratuberculosis in Switzerland. Schweiz. Arch. Fur Tierheilkd..

[35] Villaamil F.J., Yus E., Benavides B., Allepuz A., Moya S.J., Casal J., Ortega C., Diéguez F.J. (2021). Factors Associated with the Introduction of *Mycobacterium avium* spp. Paratuberculosis (MAP) into Dairy Herds in Galicia (North-West Spain): The Perception of Experts. Animals.

[36] Verteramo Chiu Leslie J., Tauer Loren W., Gröhn Yrjo T., Smith Rebecca L. (2019). Mastitis risk effect on the economic consequences of paratuberculosis control in dairy cattle: A stochastic modeling study. PLoS ONE.

[37] Thomas C.D., Lizet B.A. (2022). Mycobacterium paratuberculosis zoonosis is a One Health emergency. EcoHealth.

[38] Hernández-Bello J., Bach H., Cerpa-Cruz S., Sánchez-Zuno G.A., Hernández-Gutiérrez R., Nicoletti F., Saraceno A., Muñoz-Valle J. (2025). PtpA protein from *Mycobacterium avium* subsp. paratuberculosis as a potential marker of rheumatoid arthritis in humans. PLoS ONE.

[39] Kuenstner J.T., Zhang P., Potula R., Galarneau J.-M., Bach H. (2024). Human antibodies against *Mycobacterium avium* ssp. paratuberculosis combined with cytokine levels for the diagnosis and selection of Crohn’s disease patients for anti-mycobacterial therapy-A pilot study. PLoS ONE.

